# The correlation between muscle strength, inhibitory function, and cognitive function in older adults with cognitive impairment: evidence from resting-state electroencephalography

**DOI:** 10.3389/fnagi.2025.1574275

**Published:** 2025-06-20

**Authors:** Mi Hu, Xing Wang

**Affiliations:** ^1^Department of Physical Education, East China University of Political Science and Law, Shanghai, China; ^2^School of Physical Education, Shanghai University of Sport, Shanghai, China

**Keywords:** inhibitory function, cognitive function, muscle strength, EEG, older adult

## Abstract

**Objective:**

This study explored the associations among muscle strength, inhibitory control, and cognitive function in older adults with cognitive impairment, focusing on related EEG changes.

**Methods:**

Among 247 adults aged 70 and above, 120 with cognitive impairment were included. Assessments included the Montreal Cognitive Assessment (MoCA), Stroop task, grip strength, and resting-state EEG.

**Results:**

Inhibitory control was significantly related to cognitive function: accuracy in congruent (r = 0.599, *p* < 0.001) and incongruent (*r* = 0.474, *p* < 0.001) conditions correlated positively with MoCA scores, while response times in both conditions showed negative correlations (*r* = –0.475 and –0.354, respectively, *p* < 0.001). EEG alpha1 (C3, P3, F7), alpha2 (P3, F8), and beta1 (P3, F7) power were significantly associated with both cognitive and inhibitory performance. Muscle strength was significantly positively correlated with specific EEG indicators, particularly alpha1 power at C4 (*r* = 0.212, *p* < 0.05), O2 (*r* = 0.204, *p* < 0.05), F8 (*r* = 0.225, *p* < 0.05), and T6 (*r* = 0.206, *p* < 0.05), as well as alpha2 power at C3 (*r* = 0.216, *p* < 0.05), P3 (*r* = 0.222, *p* < 0.05), P4 (*r* = 0.268, *p* < 0.001), F8 (*r* = 0.284, *p* < 0.001), and T5 (*r* = 0.218, *p* < 0.05).

**Conclusion:**

Muscle strength may support cognitive and inhibitory function by influencing specific EEG activities. These findings highlight the neurophysiological links among muscle strength, cognition, and brain activity, offering potential biomarkers for early detection and intervention in cognitive decline.

## 1 Introduction

Population aging has become a global concern, with the incidence of neurodegenerative diseases in older adults rising steadily with age. In China, recent statistics indicate that approximately 6.0% of individuals aged 60 and above suffer from dementia, 3.9% from Alzheimer’s disease, and about 15.5% exhibit mild cognitive impairment (MCI) (Jia et al., 2020). With the accelerating pace of population aging, cognitive decline has emerged as a critical factor affecting the quality of life and independence of older adults. Among various cognitive domains, Inhibitory control—an essential component of executive function—is considered one of the most vulnerable to age-related deterioration (Vogel et al., 2024), It exhibits marked signs of deterioration in late adulthood (Jongsiriyanyong and Limpawattana, 2018). Research suggests that declines in inhibitory control not only affect an individual’s attention regulation and task performance, but are also closely associated with neurodegenerative diseases such as dementia and mild cognitive impairment (MCI) (Rabi et al., 2020). Neuroimaging studies have shown that inhibitory control primarily relies on the prefrontal cortex, particularly the integrative functions of the right dorsolateral prefrontal cortex (DLPFC) and anterior cingulate cortex (ACC), areas that are among the first and most severely affected by age-related brain degeneration (Miller and Cohen, 2001; Opwonya et al., 2022).

Currently, the diagnosis of cognitive impairment and dementia is primarily based on a combination of methods, including psychological assessments, blood tests, cerebrospinal fluid analysis, neurological examinations, and magnetic resonance imaging (MRI). Electroencephalography (EEG), a non-invasive, portable, cost-effective technique with high temporal resolution, has recently been widely used in cognitive neuroscience research and cognitive screening of older populations (Brambilla et al., 2021). Changes in EEG frequency bands reflect the load and efficiency of cognitive control: delta (0.5–4 Hz) and theta (4–7 Hz) bands are significantly activated during task conflict processing and error monitoring, Indicating the regulatory capacity of the brain’s executive system (Karamacoska et al., 2018). Studies have also found that older adults often exhibit higher delta power and weaker theta coherence during classical inhibitory tasks such as the Stroop task, suggesting that their inhibitory control may be modulated by neural oscillations at specific frequencies (Davidson et al., 2003). Additionally, the alpha band (8–12 Hz) has been widely studied in relation to inhibitory mechanisms during task processing, with higher alpha1 power (8–10 Hz) typically associated with better task performance efficiency (Rana and Vaina, 2014).

Muscle strength, as a comprehensive health indicator, is increasingly being redefined as a “predictive factor” for cognitive health (Yang et al., 2025) Several studies suggest that declines in muscle strength not only signal physical function deterioration but may also impact brain function through various neurophysiological pathways (Ma et al., 2025; Yang et al., 2024). Research has shown that skeletal muscle-derived factors produced during muscle contraction, such as brain-derived neurotrophic factor (BDNF), insulin-like growth factor 1 (IGF-1), and muscle-derived lactate, can cross the blood-brain barrier to influence the central nervous system, promoting synaptic plasticity, neurogenesis, and inflammation suppression (Kostka et al., 2024). IGF-1 is considered a key factor linking muscle strength to prefrontal cognitive function, with elevated levels enhancing synaptic density in the prefrontal cortex and improving inhibitory control efficiency within executive functions (Chen et al., 2023). Furthermore, EEG studies suggest that individuals with higher muscle strength exhibit greater low-frequency power changes in the prefrontal cortex during cognitive tasks, reflecting enhanced neural resource mobilization (Xin et al., 2025). A large-scale cross-sectional study has found a significant positive correlation between increased muscle strength and enhanced cognitive function, while reduced muscle mass is associated with greater cognitive decline in older adults (Baumgartner and Kao, 2024), In conclusion, there may be a complex and interactive relationship between muscle strength, inhibitory control, cognitive function, and their neurophysiological underpinnings. We propose that muscle health not only improves brain structure and function through the secretion of neurotrophic factors but may also regulate cognitive processing through its influence on specific EEG frequency bands. In this context, EEG could serve not only as a research tool but also as a potential biomarker for early cognitive decline risk, providing an objective basis for cognitive disorder screening and intervention.

A review of previous research indicates a close relationship between inhibitory control, cognitive function, and muscle strength. Our team’s prior study found that older adults with cognitive impairment exhibit specific EEG markers, and that muscle strength is positively correlated with cognitive function (Cai et al., 2023), based on these findings, the current study raises the following research questions: Do older adults with cognitive impairment exhibit specific EEG frequency bands associated with inhibitory control deficits? Are there overlapping EEG frequency bands related to inhibitory function and overall cognitive performance? Can muscle strength positively influence specific EEG frequency bands? Do inhibitory control and EEG frequency patterns differ by sex and levels of muscle strength in older adults? This study will employ an observational design to investigate the relationships among muscle strength, inhibitory control, cognitive function, and EEG frequency bands in older adults with cognitive impairment. By elucidating the role of EEG in these interrelations, the study aims to address the above research questions and provide evidence to inform individualized clinical assessment and intervention strategies for delaying the decline of inhibitory function in this population.

## 2 Subjects and methods

### 2.1 Subjects

The sample size for this study was estimated using G*Power software. A medium effect size of 0.35 was set, with a statistical power of 0.80 and an alpha level of 0.05. The statistical test selected was ANOVA: Fixed effects, omnibus, one-way, and the minimum required sample size was calculated to be 84 participants. Participants were recruited from four elderly service centers in Shanghai through health education sessions and recruitment posters. Using a convenience sampling method based on voluntary participation, a total of 150 older adults aged 70 and above were initially recruited. Ultimately, 107 participants were included in the final analysis.

**FIGURE 1:**
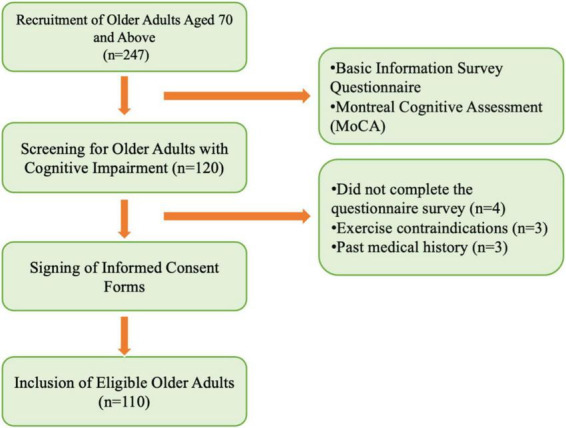
Recruitment process of study subjects.

Inclusion criteria: (1) Aged 70 years or older; (2) Right-handed; (3) In good physical condition; (4) No severe cardiovascular or cerebrovascular diseases, or major organic disorders; (5) No serious muscular diseases or contraindications to physical activity; (6) Normal vision and hearing; (7) Mentally stable, with no history of psychiatric disorders or use of psychotropic medications; (8) Able to communicate verbally and cooperate with the assessment; (9) Willing to sign the informed consent form. Exclusion criteria: (1) Participants who experienced physical discomfort leading to discontinuation during testing; (2) Participants whose behavioral or EEG data contained excessive outliers, making analysis impossible; (3) Presence of other chronic conditions that could interfere with testing. The participant recruitment process is illustrated in Figure 1.

### 2.2 Testing procedure

**FIGURE 2:**
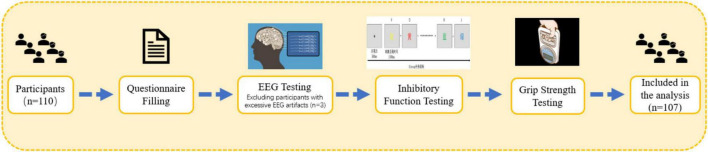
Testing procedure.

All tests were conducted between 13:30 and 16:30 on each testing day. Participants were required to visit the laboratory twice. During the first visit, the testing procedures were explained to participants, who then signed the informed consent form and completed the basic information survey, the Montreal Cognitive Assessment (MoCA), and the International Physical Activity Questionnaire-Short Form (IPAQ-SF). Height, weight, and muscle strength measurements were also taken during this visit. During the second visit, inhibitory function tasks were administered. The testing procedure is outlined in Figure 2. Participants were instructed to refrain from vigorous exercise and avoid caffeine or alcohol-containing beverages for 24 hprior to testing. All participants voluntarily participated in the study and signed the informed consent form. This study adheres to the latest version of the Declaration of Helsinki ethical standards and has received approval from the Ethics Committee of Shanghai University of Sport (102772020RT060).

### 2.3 Testing tools

#### 2.3.1 Basic information questionnaire

The basic information questionnaire includes the following items: name, age, gender, height, weight, marital status, past occupations, level of education, smoking habits, alcohol consumption, dietary habits, hearing and vision status, sleep quality, presence of exercise contraindications, medical history, and medication usage.

#### 2.3.2 Height, weight, and muscle strength testing

Height was measured using a stadiometer. Participants stood barefoot on the base plate of the stadiometer with their backs against the vertical column, keeping the torso naturally upright, the head straight, and the eyes looking forward. The measurement was recorded in centimeters to one decimal place. Weight was measured using a weighing scale. Participants stood barefoot at the center of the scale in a natural and stable posture. The measurement was recorded in kilograms to one decimal place. Height and weight were each measured twice, and the average was calculated. Body mass index (BMI) was calculated by dividing weight (in kilograms) by the square of height (in meters) using the formula: BMI = weight (kg)/height^2^ (m).

Muscle strength was assessed using a grip strength test. Participants stood upright with their arms extended naturally at their sides, holding a handgrip dynamometer. They squeezed the handle with maximum effort for 3–5 s. The test was repeated three times with a 30-sinterval between trials, and the highest grip strength value of the dominant hand was recorded. Low muscle strength was defined as a grip strength < 28 kg for men and < 18 kg for women (Chen et al., 2014).

#### 2.3.3 Montreal cognitive assessment (MoCA)

This assessment is widely used to evaluate cognitive function in older adult and includes eight domains: visuospatial/executive function, naming, memory, attention, language, abstraction, delayed recall, and orientation. The total score is 30 points, with higher scores indicating better cognitive performance. A total MoCA score of 26 or higher is considered normal, between 18 and 26 indicates mild cognitive impairment, between 10 and 17 indicates moderate impairment, and a score below 10 is classified as severe. To account for the influence of education, one point is added to the total score if the participant has 12 or fewer years of education; however, if this results in a score above 30, no additional points are given. The retest reliability of the MoCA scale used in this study is 0.857 (Xin et al., 2025).

In this study, participants were categorized into three groups based on their Montreal Cognitive Assessment (MoCA) scores. Initially, all participants’ MoCA scores were collected, and the first quartile (Q1) and third quartile (Q3) of these scores were calculated. Participants were subsequently grouped using the quartile method as follows: those with MoCA scores less than or equal to Q1 were assigned to the low cognitive function group; those with scores greater than Q1 but less than Q3 were placed in the moderate cognitive function group; and those with scores greater than or equal to Q3 were placed in the high cognitive function group. This quartile grouping method facilitates a more effective comparison of the impact of different cognitive function levels on various research indicators.

#### 2.3.4 Stroop task

Inhibitory function is assessed using the Stroop task paradigm. The task consists of two phases: a practice phase followed by a formal experimental phase. The practice phase comprises 20 trials, with feedback provided to participants regarding accuracy and reaction time after each trial. The formal experiment begins once participants achieve a 75% accuracy rate during the 20 practice trials. If this requirement is not met, the practice phase continues, with no more than three attempts allowed. After the practice phase, participants rest for 30 s before beginning the formal phase. The stimuli include the Chinese characters “red,” “yellow,” “blue,” and “green,” each presented in its respective color. A total of 16 stimuli are presented, with four congruent and 12 incongruent. The font size remains consistent, and stimuli are presented in random order. After the fixation point “+” appears, a colored word is displayed at the center of the screen. The formal experiment consists of a single block. In the congruent condition, participants identify the color of the target word. Participants press the “D” key for red, “F” for yellow, “J” for blue, and “K” for green, with a total of 24 trials. In the incongruent condition, participants identify the color of the target word. Participants press the corresponding key based on the color of the word: “D” for red, “F” for yellow, “J” for blue, and “K” for green. The stimulus is presented for 1,500 ms, with a total of 48 trials.

#### 2.3.5 EEG Signal acquisition and processing

The testing was conducted in an indoor environment that was soundproof, well-ventilated, and had appropriate lighting, temperature, and humidity, with no electromagnetic interference. Prior to testing, participants were instructed to wash and dry their hair. They were seated comfortably in an upright position. The experimenter instructed participants to keep their eyes closed, remain relaxed, quiet, and awake, and to avoid blinking, teeth clenching, swallowing, and body movements. Electroencephalogram (EEG) signals were recorded using the NCERP-190012 EEG system (Shanghai Nuocheng Electric Co., Ltd.). Sixteen conductive electrodes were placed according to the international 10–20 system: Fp1 (left prefrontal), Fp2 (right prefrontal), F3 (left frontal), F4 (right frontal), C3 (left central), C4 (right central), P3 (left parietal), P4 (right parietal), O1 (left occipital), O2 (right occipital), F7 (left frontal lateral), F8 (right frontal lateral), T3 (left mid-temporal), T4 (right mid-temporal), T5 (left posterior temporal), and T6 (right posterior temporal). The ground electrode was placed at the frontal midline (GND), and reference electrodes were placed on the bilateral mastoids (A1 and A2). The device was equipped with a preamplifier, with the sampling rate set at 500 Hz, high-pass filter at 0.3 Hz, low-pass filter at 30 Hz, and electrode impedance maintained below 5 kΩ.EEG signals were divided into six frequency bands: delta (1–4 Hz), theta (4–8 Hz), alpha1 (8–10.5 Hz), alpha2 (10.5–13 Hz), beta1 (13–20 Hz), and beta2 (20–30 Hz). Resting-state EEG was recorded for 5 min for each participant. Data preprocessing was performed using the EEGLAB toolbox in MATLAB 2023b. First, data were re-referenced and band-pass filtered between 0.1–30 Hz to remove low-frequency drifts and high-frequency electromyographic noise. After preliminary filtering, segments with obvious artifacts (e.g., electrode drop-out or large amplitude fluctuations) were manually inspected and removed. The rejection criterion was an amplitude exceeding ± 100 μV or the presence of abnormal waveform patterns. To further eliminate ocular and muscle artifacts, Independent Component Analysis (ICA) using the Infomax algorithm was applied to isolate components related to eyelid movements, eye rotation, and facial muscle activity. These components were identified and removed based on their scalp distribution, temporal characteristics, and spectral features. After artifact rejection, data quality was further assessed. Participants whose EEG data had more than 5% of channels contaminated by residual artifacts were excluded. To ensure data integrity and comparability, only participants with at least 80% usable EEG data were included in the final statistical analysis.

### 2.4 Statistical analysis

Data were statistically analyzed using SPSS 29.0 software. Continuous data were expressed as mean ± standard deviation, with results presented to two decimal places. Between-group comparisons were conducted using one-way analysis of variance (ANOVA). Categorical data were described using frequencies (n), with between-group comparisons performed using the χ2 test. Pearson correlation analysis was used to explore the relationships between muscle strength and inhibitory function, cognitive function, and EEG indicators. One-way ANOVA and LSD *post hoc* tests were used to compare differences in inhibitory function and specific EEG indicators between older adults of different genders and muscle strength levels. Given the multiple correlation analyses conducted in this study, Bonferroni correction was applied to control for Type I error. For the correlation analyses among inhibitory control, cognitive function scores, and muscle strength, the adjusted significance level was set at α = 0.0125. For the correlation analyses between inhibitory control, cognitive function scores, and EEG indicators across six frequency bands, the corrected significance level was α≈0.0083. For the correlation analyses between muscle strength and specific EEG frequency bands, the corrected threshold was α = 0.0167. Between-group differences were assessed using LSD *post hoc* multiple comparisons.

## 3 Results

### 3.1 Differences in baseline information and inhibitory function among older adults with varying cognitive function scores

Older adults were screened using the Montreal Cognitive Assessment (MoCA) and electroencephalography (EEG). 3 participants were excluded due to substantial EEG artifacts caused by external interference. A total of 107 older adults were ultimately included in the study. The mean age of the included participants was 73.673 ± 7.669 years. Significant differences were observed among groups with varying levels of cognitive impairment in terms of age, marital status, previous occupation, educational attainment, alcohol consumption, and measures of inhibitory control (*p* < 0.05). No significant differences were found in other variables (*p* > 0.05). For detailed results (Table 1).

**TABLE 1:** Comparison of baseline information and inhibitory function among older adults with different cognitive function scores.

Variables		Cognitive function score classification			Difference test
	**Overall (107)**	**Mild (*n* = 39)**	**Moderate (*n* = 34)**	**Severe (*n* = 34)**	
Age (years)	73.67 ± 7.669	70.36 ± 6.417	72.47 ± 6.106	78.68 ± 7.995	*F* = 14.086, P < 0.001
BMI kg/m^2^	24.023 ± 3.541	24.906 ± 3.354	23.928 ± 3.452	23.023 ± 3.688	*F* = 2.431, P = 0.093
Gender					*X*^2^ = 0.979, P = 0.613
Male	70	27	20	23	
Female	37	12	14	11	
Marital Status					*X*^2^ = 9.066, P = 0.011
Married	87	35	30	22	
Unmarried, divorced or Widowed	20	4	4	12	
Past occupations					*X*^2^ = 8.213, P = 0.016
Farmer	72	20	24	28	
Non-farmer	35	19	10	6	
Level of education					*X*^2^ = 21.257, P = 0.002
Illiterate	21	0	8	13	
Primary school	60	24	18	18	
Junior high school	21	12	6	3	
High school and above	5	3	2	0	
Smoking habits					*X*^2^ = 2.452, P = 0.293
Smoker	47	21	13	13	
Non-smoker	60	18	21	21	
Alcohol consumption					*X*^2^ = 6.429, P = 0.040
Drinker	48	23	15	10	
Non-drinker	59	16	19	24	
Dietary habits					*X*^2^ = 5.840, P = 0.211
Vegetarian	38	10	12	16	
Meat-based diet	6	3	3	0	
Mixed diet	63	26	19	18	
Hearing status					*X*^2^ = 4.114, P = 0.128
Declined	55	15	20	20	
Normal	52	24	14	14	
Vision status					*X*^2^ = 1.991, P = 0.369
Declined	72	24	26	22	
Normal	35	25	8	12	
Sleep quality					*X*^2^ = 2.519, P = 0.866
Very good	13	3	5	5	
Good	68	25	21	22	
Fair	24	10	8	6	
Poor	2	1	0	1	
Congruent condition accuracy rate	0.91 ± 0.17	0.97 ± 0.05	0.97 ± 0.05	0.78 ± 0.23	*F* = 23.639, *P* < 0.001
Congruent condition reaction time	1.18 ± 0.26	1.07 ± 0.19	1.13 ± 0.18	1.36 ± 0.32	*F* = 14.774, *P* < 0.001
Incongruent condition accuracy Rate	0.90 ± 0.18	0.95 ± 0.10	0.96 ± 0.07	0.78 ± 0.25	*F* = 14.637, *P* < 0.001
Incongruent condition reaction time	1.27 ± 0.27	1.20 ± 0.24	1.21 ± 0.23	1.40 ± 0.31	*F* = 6.894, *P* < 0.001

*Post hoc* multiple comparisons of inhibitory control measures revealed that older adults with severe cognitive impairment had significantly lower accuracy and longer reaction times compared to those with mild to moderate cognitive impairment. These findings suggest that greater age is associated with increased severity of cognitive impairment. Moreover, older adults who are unmarried, divorced, or widowed are more likely to suffer from severe cognitive impairment. Individuals with a farming background are at higher risk of developing cognitive impairment across all severity levels compared to those from non-farming backgrounds. Higher educational attainment appears to be protective against cognitive impairment. Overall, older adults with cognitive impairment exhibit a decline in inhibitory control, with those in the severe group showing markedly poorer performance than those with mild to moderate impairment.

### 3.2 Relationship between inhibitory function and cognitive function scores in older adults with cognitive impairment

Pearson correlation coefficients were used to examine the relationship between inhibitory function and cognitive function (Table 2), revealing a significant association between these variables (*P* < 0.001). Correlation analyses were conducted between the four indices of inhibitory control (i.e., congruent condition accuracy rate, congruent condition reaction time, incongruent condition accuracy rate, and incongruent condition reaction time) and cognitive function scores. A total of four tests were performed, and the Bonferroni correction was applied to adjust for multiple comparisons. The adjusted significance level was set at α = 0.05/4 = 0.0125.Specifically, congruent condition accuracy (*r* = 0.599, *P* < 0.001) and incongruent condition accuracy (*r* = 0.474, *P* < 0.001) were significantly positively correlated with MoCA scores, indicating that higher MoCA scores were associated with greater accuracy in both congruent and incongruent conditions. Congruent condition reaction time (*r* = −0.475, *P* < 0.001) and incongruent condition reaction time (*r* = −0.354, *P* < 0.001) were significantly negatively correlated with MoCA scores, indicating that higher MoCA scores were associated with faster reaction times in both conditions. These correlations suggest that inhibitory function is indicative of overall cognitive function, and enhancing inhibitory function may positively influence cognitive outcomes.

**TABLE 2:** Correlations between Inhibitory control and MoCA scores.

	MoCA	Congruent condition accuracy rate	Congruent condition reaction time	Incongruent condition accuracy rate	Incongruent condition reaction time
MoCA	1				
Congruent condition accuracy rate	0.599**	1			
Congruent condition reaction time	−0.475**	−0.544**	1		
Incongruent condition accuracy rate	0.474**	0.871**	−0.573**	1	
Incongruent condition reaction time	−0.354**	−0.419**	0.844**	−0.426**	1

**p < 0.001; *p < 0.05.

### 3.3 Correlation between cognitive function scores and EEG indicators in older adults with cognitive impairment

Pearson correlation analysis was conducted to examine the relationship between MoCA scores and EEG indicators in older adults with cognitive impairment (Table 3). Correlation analyses were conducted between cognitive function scores and each of the six EEG frequency bands. Bonferroni correction was applied, and the adjusted significance level was set at α = 0.05/6 ≈ 0.0083. The results revealed significant negative correlations between the MoCA scores and the delta, theta, and beta2 bands in FP1 and FP2, as well as the beta2 band in T3. Specifically, higher MoCA scores were associated with lower activation levels of these EEG indicators. In contrast, significant positive correlations were found between the MoCA scores and the delta band in T4, as well as the alpha1 and alpha2 bands across the whole brain and the beta1 band in C3, C4, P3, P4, F7, and F8. Higher MoCA scores were associated with higher activation levels of these EEG indicators.

**TABLE 3:** The correlation between MoCA Scores and EEG indices.

	Delta	Theta	Alpha1	Alpha2	Beta1	Beta2
FP1	-0.296**	-0.348**	0.363**	0.258**	0.052	-0.210*
FP2	-0.244*	-0.295**	0.391**	0.248*	0.070	-0.221*
F3	-0.123	-0.168	0.379**	0.251**	0.039	-0.184
F4	-0.051	-0.102	0.409**	0.292**	0.137	-0.055
C3	0.010	-0.091	0.415**	0.361**	0.203*	-0.046
C4	-0.049	-0.106	0.407**	0.352**	0.244*	0.017
P3	0.028	-0.073	0.406**	0.364**	0.227*	0.068
P4	-0.002	-0.079	0.404**	0.366**	0.281**	0.084
O1	0.022	-0.115	0.422**	0.340**	0.130	-0.096
O2	-0.026	-0.170	0.392**	0.329**	0.186	-0.053
F7	0.037	-0.075	0.421**	0.375**	0.243*	0.050
F8	0.022	-0.060	0.414**	0.369**	0.274**	0.079
T3	-0.044	-0.184	0.265**	0.159	-0.093	-0.259**
T4	0.201*	0.086	0.354**	0.307**	0.060	-0.122
T5	0.020	-0.096	0.398**	0.338**	0.133	-0.160
T6	-0.039	-0.172	0.391**	0.324**	0.185	-0.088

**p < 0.001;

*p < 0.05.

These findings suggest that lower activation levels of delta, theta, and beta2 in the prefrontal cortex are associated with better cognitive performance, indicating that activation of these EEG indicators in the resting state may contribute to cognitive decline in older adults. The significant positive correlations between MoCA scores and delta in T4, as well as alpha1, alpha2, and beta1 in various brain regions, suggest that older adults with higher cognitive function exhibit higher EEG activity, particularly in the parietal and frontal regions.

### 3.4 Correlation between inhibitory function and EEG indicators in older adults with cognitive impairment

#### 3.4.1 Correlation coefficients between consistency accuracy and EEG indicators

Correlation analyses were performed between accuracy in the congruent condition and each of the six EEG frequency bands. Bonferroni correction was applied, and the adjusted significance level was set at α = 0.05/6≈0.0083. The results in Table 4 reveal that the delta and theta bands in FP1, as well as the theta band in FP2, show significant negative correlations with accuracy under consistent conditions (*P* < 0.05). Specifically, higher consistency accuracy is associated with lower activation levels of these EEG indicators. In contrast, the delta band in O1, F7, and T5, as well as alpha1 across the whole brain, and the alpha2 bands in C3, C4, P3, P4, O1, O2, F7, F8, T3, T4, T5, and T6, as well as the beta1 power in C3, P3, P4, F7, and F8, are significantly positively correlated with accuracy under consistent conditions (*P* < 0.05). In this case, higher consistency accuracy is associated with higher activation levels of these EEG indicators.

**TABLE 4:** Correlation coefficients between congruent accuracy rate and EEG power values.

	Delta	Theta	Alpha1	Alpha2	Beta1	Beta2
FP1	-0.195*	-0.236*	0.276**	0.182	0.157	-0.083
FP2	-0.132	-0.192*	0.252**	0.136	0.040	-0.169
F3	0.027	-0.094	0.290**	0.189	0.106	-0.075
F4	0.055	-0.055	0.276**	0.176	0.054	-0.013
C3	0.158	-0.002	0.353**	0.309**	0.236*	0.063
C4	0.096	-0.020	0.316**	0.259**	0.189	0.040
P3	0.189	0.024	0.351**	0.316**	0.254**	0.177
P4	0.129	0.002	0.326**	0.287**	0.238*	0.086
O1	0.201*	-0.039	0.323**	0.274**	0.164	0.028
O2	0.124	-0.045	0.307**	0.252**	0.174	-0.001
F7	0.191*	0.016	0.357**	0.326**	0.268**	0.158
F8	0.154	0.024	0.328**	0.289**	0.234*	0.083
T3	0.027	-0.149	0.216*	0.134	0.011	-0.176
T4	0.138	-0.098	0.258**	0.204*	0.008	-0.117
T5	0.204*	-0.012	0.317**	0.284**	0.173	-0.056
T6	0.104	-0.053	0.311**	0.258**	0.181	-0.024

** *p* < 0.001;

* *p* < 0.05.

#### 3.4.2 Correlation between reaction time under consistent conditions and EEG indicators

Correlation analyses were conducted between response time in the congruent condition and each of the six EEG frequency bands. Bonferroni correction was applied, with the adjusted significance level set at α = 0.05/6 ≈ 0.0083. The results in Table 5 show that the delta band in C3, P3, F7, and T5, the alpha1 band in C3, C4, P3, P4, O1, O2, F7, F8, T5, and T6, the alpha2 band in C3, P3, P4, F8, and T5, and the beta1 band in P3, P4, F7, and F8 are significantly negatively correlated with reaction time under consistent conditions (*P* < 0.05). Specifically, longer reaction times under consistent conditions are associated with lower activation levels of these EEG indicators. On the other hand, the theta band in FP1, the beta1 band in T3, and the theta bands in FP1, FP2, F3, T3, and T4 show significant positive correlations with reaction time under consistent conditions (*P* < 0.05). In this case, longer reaction times are associated with higher activation levels of these EEG indicators.

**TABLE 5:** Correlation coefficients between congruent reaction time and EEG power values.

	Delta	Theta	Alpha1	Alpha2	Beta1	Beta2
FP1	0.030	0.203*	-0.158	-0.040	0.018	0.213*
FP2	0.040	0.161	-0.159	-0.034	0.054	0.206*
F3	-0.021	0.167	-0.181	-0.057	0.018	0.194*
F4	-0.011	0.069	-0.179	-0.077	0.005	0.019
C3	-0.191*	-0.021	-0.297**	-0.204*	-0.154	0.008
C4	-0.105	0.003	-0.269**	-0.177	-0.127	0.034
P3	-0.224*	-0.067	-0.317**	-0.244*	-0.209*	-0.102
P4	-0.138	-0.036	-0.310**	-0.224*	-0.207*	-0.065
O1	-0.175	0.006	-0.264**	-0.150	-0.079	0.051
O2	-0.079	0.055	-0.274**	-0.180	-0.119	0.123
F7	-0.218*	-0.048	-0.324**	-0.247*	-0.223*	-0.042
F8	-0.173	-0.072	-0.316**	-0.241*	-0.228*	-0.098
T3	-0.011	0.137	-0.049	0.031	0.205*	0.374**
T4	-0.068	0.070	-0.147	-0.144	0.070	0.196*
T5	-0.193*	-0.045	-0.285**	-0.190*	-0.107	0.175
T6	-0.074	0.051	-0.289**	-0.188	-0.113	0.139

**p < 0.001;

* *p* < 0.05.

#### 3.4.3 Correlation between inhibitory function under inconsistent conditions and EEG indicators

Correlation analyses were conducted between accuracy in the incongruent condition and each of the six EEG frequency bands. Bonferroni correction was applied, with the adjusted significance level set at α = 0.05/6 ≈ 0.0083. The results in Table 6 show that the delta band in C3, P3, O1, F7, F8, and T5, the alpha1 band across the whole brain, the alpha2 band in FP1, F3, C3, C4, P3, P4, O1, O2, F7, F8, T4, T5, T6, and the beta1 band in FP1, C3, P3, P4, O1, F7, F8, and T5, as well as the beta2 band in P3 and F7, are significantly positively correlated with correct response rates under inconsistent conditions (P < 0.05). Specifically, higher correct response rates under inconsistent conditions are associated with higher activation levels of these EEG indicators.

**TABLE 6:** Correlation coefficients between incongruent accuracy rate and EEG power values.

	delta	theta	alpha1	alpha2	beta1	beta2
FP1	-0.095	-0.128	0.222*	0.197*	0.216*	0.016
FP2	-0.041	-0.093	0.198*	0.133	0.061	-0.114
F3	0.097	0.002	0.232*	0.203*	0.171	0.029
F4	0.132	0.035	0.219*	0.168	0.072	0.018
C3	0.202*	0.079	0.293**	0.297**	0.256**	0.129
C4	0.157	0.067	0.254**	0.235*	0.176	0.052
P3	0.226*	0.101	0.293**	0.301**	0.259**	0.213*
P4	0.180	0.085	0.261**	0.253**	0.210*	0.097
O1	0.240*	0.059	0.271**	0.271**	0.190*	0.082
O2	0.179	0.049	0.248**	0.225*	0.153	0.014
F7	0.231*	0.096	0.298**	0.307**	0.269**	0.205*
F8	0.199*	0.100	0.264**	0.257**	0.206*	0.094
T3	0.162	0.014	0.234*	0.186	0.076	-0.087
T4	0.164	-0.054	0.241*	0.212*	0.032	-0.083
T5	0.246*	0.080	0.268**	0.287**	0.203*	0.028
T6	0.162	0.042	0.253**	0.232*	0.165	0.004

**p < 0.001;

*p < 0.05.

#### 3.4.4 Correlation between inhibitory function under inconsistent response time and EEG indicators in older adults with cognitive impairment

Correlation analyses were conducted between reaction time in the incongruent condition and each of the six EEG frequency bands. Bonferroni correction was applied, with the adjusted significance level set at α = 0.05/6≈0.0083. The results in Table 7 show that the delta band in C3, P3, F7, the alpha1 band in F3, C3, C4, P3, P4, O1, O2, F7, F8, T5, T6, the alpha2 band in C3, P3, P4, F7, F8, and the beta1 band in P3 and P4 are significantly negatively correlated with response time under inconsistent conditions. Specifically, longer response times under inconsistent conditions are associated with lower activation levels of these EEG indicators. The beta1 band in T3, as well as the beta2 bands in T3 and T4, are significantly positively correlated with response time under inconsistent conditions, indicating that longer response times are associated with higher activation levels of these EEG indicators.

**TABLE 7:** Correlation coefficients between incongruent reaction time and EEG power values.

	delta	theta	alpha1	alpha2	beta1	beta2
FP1	0.054	0.203*	-0.165	-0.032	-0.009	0.145
FP2	0.062	0.160	-0.155	-0.003	0.070	0.160
F3	-0.014	0.160	-0.203*	-0.065	-0.020	0.141
F4	0.003	0.068	-0.181	-0.049	0.026	0.041
C3	-0.201*	-0.031	-0.308**	-0.200*	-0.177	-0.035
C4	-0.111	-0.005	-0.278**	-0.156	-0.106	0.032
P3	-0.238*	-0.073	-0.327**	-0.244*	-0.232*	-0.129
P4	-0.152	-0.045	-0.319**	-0.215*	-0.204*	-0.090
O1	-0.169	0.006	-0.270**	-0.149	-0.106	0.025
O2	-0.090	0.035	-0.280**	-0.161	-0.106	0.113
F7	-0.221*	-0.052	-0.327**	-0.242*	-0.237*	-0.075
F8	-0.170	-0.072	-0.321**	-0.225*	-0.220*	-0.111
T3	0.017	0.158	-0.046	0.039	0.209*	0.307**
T4	-0.065	0.045	-0.123	-0.082	0.157	0.226*
T5	-0.189	-0.039	-0.297**	-v0.183	-0.125	0.106
T6	-0.094	0.023	-0.295**	-0.173	-0.099	0.109

**p < 0.001;

*p < 0.05.

The results indicate that the delta band in F7 and T5, the alpha1 band in C3, C4, P3, P4, O1, O2, F7, F8, T5, T6, the alpha2 band in C3, P3, P4, F8, T5, and the beta1 band in P3, F7, F8 are significantly correlated with inhibitory function (P < 0.05), and serve as EEG-specific indicators for inhibitory function. This suggests that these EEG indicators may play an important role in inhibitory function. The delta band in F7 and T5, as well as alpha1, alpha2, and beta1 bands across several brain regions, are significantly correlated with inhibitory function. These specific EEG indicators may play a crucial role in inhibitory function, offering potential biomarkers for the identification and diagnosis of inhibitory dysfunction.

The results of the correlation between EEG indicators, inhibitory function, and cognitive function show that the EEG indicators shared by both inhibitory and cognitive functions include the alpha1 band in C3, C4, P3, P4, O1, O2, F7, F8, T5, T6, the alpha2 band in C3, P3, P4, F8, T5, and the beta1 band in P3, F7, F8. These EEG indicators exhibit significant correlations with both inhibitory function and cognitive function, highlighting the key brain regions and frequency bands involved in both functions.

### 3.5 The relationship between muscle strength and inhibitory function in older adults with cognitive impairment

Pearson correlation analysis was used to examine the relationship between muscle strength and inhibitory function (Table 8). Correlation analyses were conducted between muscle strength and the four indicators of inhibitory function (i.e., congruent condition accuracy rate, congruent condition reaction time, incongruent condition accuracy rate, and incongruent condition reaction time). A total of four tests were performed, and Bonferroni correction was applied, with the adjusted significance level set at α = 0.05/4 = 0.0125. The results showed that muscle strength was significantly positively correlated with correct response rates in both congruent (*r* = 0.296, *P* < 0.001) and incongruent conditions (*r* = 0.240, *P* < 0.05), indicating that higher muscle strength was associated with higher accuracy in both conditions. Additionally, muscle strength was significantly negatively correlated with response times in both congruent (*r* = −0.379, *P* < 0.001) and incongruent conditions (*r* = −0.317, *P* < 0.001), suggesting that higher muscle strength was associated with shorter response times in both conditions. These results indicate that older adults with higher muscle strength tend to have higher accuracy in inhibitory function tasks. This suggests that enhancing muscle strength may be related to improved inhibitory capacity. The negative correlation between muscle strength and response times in both congruent and incongruent conditions implies that older adults with greater muscle strength tend to exhibit faster reaction times in inhibitory function tests.

**TABLE 8:** Relationship between muscle strength and inhibitory control in older adults.

	Muscle Strength	Congruent condition accuracy rate	Congruent condition reaction time	Incongruent condition accuracy rate	Incongruent condition reaction time
Muscle strength	1				
Congruent condition accuracy rate	0.296**	1			
Congruent condition reaction time	−0.379**	−0.544**	1		
Incongruent condition accuracy rate	0.240*	0.871**	−0.573**	1	
Incongruent condition reaction time	−0.317**	−0.419**	0.884**	−0.426**	1

**p < 0.001;

*p < 0.05.

### 3.6 The Correlation between muscle strength and EEG-specific indicators in older adults with cognitive impairment

**FIGURE 3:**
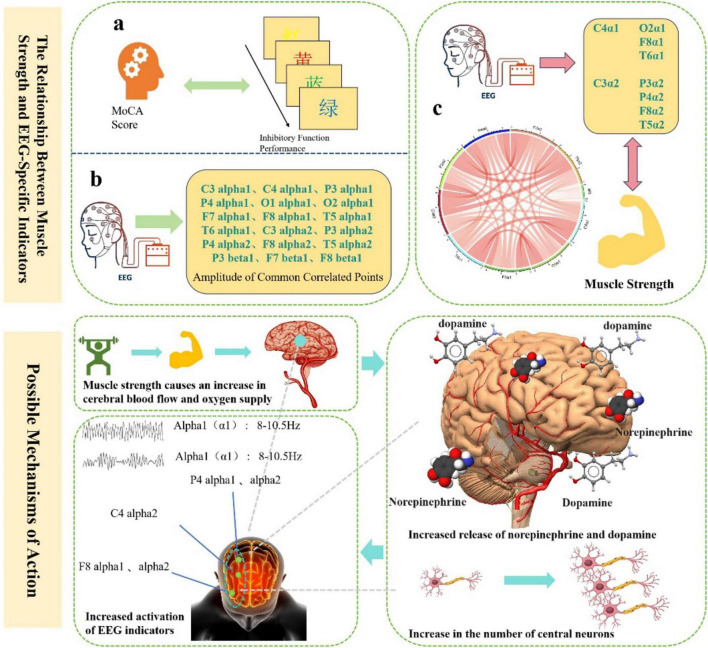
The relationship between muscle strength and EEG-Specific indicators and possible mechanisms of action.

We further explored the correlation between muscle strength and EEG indicators related to inhibitory function. Correlation analyses were conducted between muscle strength and four specific EEG frequency bands. Bonferroni correction was applied, with the adjusted significance level set at α = 0.05/3 = 0.0167. As shown in Table 9, muscle strength was significantly positively correlated with alpha1 power at C4 (*r* = 0.212, *p* < 0.05), O2 (*r* = 0.204, *p* < 0.05), F8 (*r* = 0.225, *p* < 0.05), and T6 (*r* = 0.206, *p* < 0.05), as well as with alpha2 power at C3 (*r* = 0.216, *p* < 0.05), P3 (*r* = 0.222, *p* < 0.05), P4 (*r* = 0.268, *p* < 0.001), F8 (*r* = 0.284, *p* < 0.001), and T5 (*r* = 0.218, *p* < 0.05). Higher muscle strength was associated with greater activation in these EEG regions. These results suggest that muscle strength is related to neurophysiological activity, and higher muscle strength may enhance the activation of the associated EEG indicators (Figure 3).

**TABLE 9:** Correlation coefficients between muscle strength and EEG specific indexes.

	Alpha1	Alpha2	Beta1
C3	0.152	0.216*	NA
C4	0.212*	NA	NA
P3	0.160	0.222*	0.100
P4	NA	0.268**	NA
O1	0.161	NA	NA
O2	0.204*	NA	NA
F7	0.171	NA	0.113
F8	0.225*	0.284**	0.171
T5	0.158	0.218*	NA
T6	0.206*	NA	NA

**p < 0.001;

*p < 0.05.

### 3.7 Differences in inhibitory function and EEG indicators between older adults of different genders and muscle strength

*Post hoc* multiple comparisons were conducted using the Least Significant Difference (LSD) test. The older adults of different genders were divided into normal muscle strength and low muscle strength groups. Significant differences were found among the four groups in terms of congruent accuracy, reaction time, incongruent reaction time, and the alpha1 band at C4, P4, and F8, as well as the alpha2 band at P4 and F8. Muscle strength in older adults was found to be related to the alpha1 band in the right lateral frontal, central, and parietal regions, as well as the alpha2 band in the right parietal and anterior temporal regions. In terms of congruent accuracy, the normal muscle strength group outperformed the low muscle strength group across different genders. Regarding reaction time under both congruent and incongruent conditions, the normal muscle strength group had shorter reaction times than the low muscle strength group. For the alpha1 band at C4, P4, and F8, and the alpha2 band at P4 and F8, the normal muscle strength male group exhibited higher values than the normal muscle strength female group, which was in turn higher than the low muscle strength female group, followed by the low muscle strength male group. Detailed data is presented in Table 10. The results suggest that in terms of congruent accuracy, the normal muscle strength group performed better than the low muscle strength group, indicating that higher muscle strength is associated with higher inhibitory function accuracy. In terms of reaction time under both congruent and incongruent conditions, the normal muscle strength group had shorter reaction times, which suggests that muscle strength is linked to greater inhibitory efficiency. Gender differences were observed in inhibitory function and muscle strength. The normal muscle strength male group outperformed the female group in inhibitory function, while the low muscle strength female group performed better than the low muscle strength male group. This may imply that there are gender differences in muscle strength and inhibitory function. Specific EEG indicators (such as alpha1 at C4, P4, F8, and alpha2 at P4, F8) showed significant differences across gender and muscle strength groups, suggesting that muscle strength is associated with these EEG indicators. Furthermore, the correlation between alpha1 and alpha2 in the right hemisphere and muscle strength suggests that muscle strength may influence these brain electrical activities.

**TABLE 10:** Comparative analysis of inhibition function and EEG specific indicators in elderly people of different genders and muscle strength groups.

Variable	Muscle strength (Male)		Muscle strength (Female)		*F*	*P*
	**Low (*n* = 35)**	**Normal (*n* = 35)**	**Low (*n* = 19)**	**Normal (*n* = 18)**		
Congruent condition accuracy rate	0.87 @ 0.20	0.94 @ 0.12	0.85 @ 0.21	0.99 @ 0.02	3.467	0.019
Congruent condition reaction time	1.32 @ 0.29	1.10 @ 0.20	1.26 @ 0.25	0.98 @ 0.14	10.554	0.000
Incongruent condition accuracy rate	0.87 @ 0.20	0.91 @ 0.15	0.86 @ 0.24	0.98 @ 0.04	2.047	0.112
Incongruent condition reaction time	1.37 @ 0.27	1.22 @ 0.26	1.35 @ 0.28	1.07 @ 0.15	7.031	0.000
F7 delta	3.30 @ 1.33	3.11 @ 0.88	3.81 @ 1.46	3.48 @ 0.83	1.591	0.196
T5 delta	1.83 @ 0.59	1.73 @ 0.56	2.09 @ 0.81	1.98 @ 0.45	1.785	0.155
C3 alpha1	11.96 @ 5.34	16.21 @ 8.83	14.75 @ 7.63	16.27 @ 9.57	2.125	0.102
C4 alpha1	11.69 @ 5.62	17.72 @ 11.28	14.69 @ 7.38	14.95 @ 8.81	2.836	0.042
P3 alpha1	18.38 @ 8.50	25.49 @ 15.24	23.23 @ 12.53	25.60 @ 14.39	2.221	0.090
P4 alpha1	17.58 @ 9.29	27.07 @ 17.93	22.75 @ 11.48	23.59 @ 13.28	2.859	0.041
O1 alpha1	10.42 @ 3.94	14.07 @ 8.16	12.68 @ 6.71	13.57 @ 6.48	2.056	0.111
O2 alpha1	10.43 @ 4.96	15.06 @ 9.58	12.72 @ 6.08	13.09 @ 6.43	2.435	0.069
F7 alpha1	18.27 @ 8.49	25.81 @ 14.97	23.39 @ 12.34	25.60 @ 14.39	2.483	0.065
F8 alpha1	17.64 @ 9.01	27.38 @ 17.72	22.86 @ 11.37	23.59 @ 13.28	3.071	0.031
T5 alpha1	10.23 @ 3.91	13.76 @ 8.44	12.45 @ 6.94	13.57 @ 6.48	1.924	0.130
T6 alpha1	10.17 @ 4.93	14.91 @ 9.63	12.54 @ 6.24	13.09 @ 6.43	2.538	0.061
C3 alpha2	17.63 @ 5.35	21.88 @ 10.19	18.87 @ 5.89	21.13 @ 5.80	2.200	0.093
P3 alpha2	27.21 @ 9.89	34.97 @ 17.48	30.04 @ 10.84	34.01 @ 9.87	2.394	0.073
P4 alpha2	26.03 @ 10.88	35.61 @ 18.70	29.65 @ 9.28	31.58 @ 9.93	2.945	0.036
F8 alpha2	26.12 @ 10.59	35.74 @ 18.52	29.36 @ 9.82	31.58 @ 9.93	3.038	0.032
T5 alpha2	16.19 @ 4.50	19.66 @ 9.17	16.84 @ 6.16	19.15 @ 5.19	1.910	0.133
P3 beta1	27.91 @ 9.42	30.61 @ 11.86	29.33 @ 10.82	32.63 @ 9.39	0.893	0.448
F7 beta1	27.69 @ 9.76	30.83 @ 11.45	29.31 @ 10.85	32.63 @ 9.39	1.036	0.380
F8 beta1	26.35 @ 9.68	30.27 @ 11.58	27.92 @ 8.35	29.98 @ 10.04	1.015	0.389

## 4 Discussion

This study found that an individual’s performance in inhibitory tasks is closely related to their overall cognitive function level. Specifically, accuracy in both congruent and incongruent conditions showed a significant positive correlation with the total MoCA score, while reaction time demonstrated a significant negative correlation with the MoCA total score. This result supports previous findings, indicating that individuals with higher cognitive function tend to exhibit greater accuracy and faster responses in inhibitory tasks (Nashiro et al., 2023; Yang Y. et al., 2020). From a neuropsychological perspective, cognitive function is a multidimensional construct encompassing attention, memory, executive function, language, and visuospatial abilities (Yang X. et al., 2020). In this context, inhibitory control, as a core component of executive function, primarily involves the inhibition of automatic responses, conflict monitoring, and selective attention processes (Vogel et al., 2024), its neural basis mainly involves the regulation of areas in the prefrontal cortex, particularly the dorsolateral prefrontal cortex (DLPFC) and anterior cingulate cortex (ACC) (Tozzi et al., 2020). These brain regions play a crucial role in various dimensions assessed by the MoCA, which explains the significant correlation between inhibitory task performance and the MoCA total score. Specifically, the accuracy and reaction time observed in congruent and incongruent conditions reflect not only differences in participants’ processing speed and conflict resolution abilities but also their efficiency in allocating attention resources and cognitive control (Hassel et al., 2020). Higher MoCA scores are typically associated with better executive function, enabling individuals to more effectively manage the cognitive demands in conflict conditions and achieve better performance on inhibitory tasks. This finding further validates the foundational role of inhibitory control within cognitive structure (Dallaway et al., 2023). Based on these findings, it is suggested that clinical practitioners and researchers, when designing future intervention studies, explore treatment strategies centered on inhibitory training. Such approaches may significantly enhance comprehensive cognitive function indicators like MoCA and offer theoretical and practical support for delaying cognitive decline or promoting cognitive recovery.

This study systematically revealed the relationship between cognitive function, inhibitory control, and resting-state EEG power in various frequency bands through correlation analysis, further clarifying the role of specific brain regions and frequency bands in the mechanisms underlying cognitive impairment in older adults. Specifically, delta, theta, and beta2 power values in the frontal lobe regions (FP1, FP2) showed significant negative correlations with MoCA scores, suggesting that abnormal increases in low and some mid-frequency brain waves during rest may reflect inefficiency in the frontal cortex’s cognitive integration and monitoring functions (Aminov et al., 2017). Particularly, delta waves (1–4 Hz), typically associated with cortical inhibition and neural synchronization, are often considered an early marker of neurodegenerative changes when enhanced in a waking state (Bernat et al., 2015). The excessive activation of theta waves (4–8 Hz) in the frontal lobe may result from “over-processing” of executive function resources in the brain (Bernat et al., 2015), and abnormal activation during task-free resting states may reflect compensatory disruption in the prefrontal-hippocampal neural circuit (Wang et al., 2015). In contrast, delta waves in the T4 region, and alpha1 (8–10 Hz) and alpha2 (10–13 Hz) waves across the brain, along with beta1 (13–20 Hz) in the parietal (P3, P4), central (C3, C4), and frontal (F7, F8) areas, showed positive correlations with MoCA scores. This phenomenon suggests that the enhancement of alpha and beta1 waves may reflect more efficient neural regulation mechanisms. Studies have already shown that alpha waves represent the brain’s capacity for information inhibition and regulation, with increased power being closely associated with better attention allocation, perceptual selection, and task preparation (Gutteling et al., 2022). The enhanced alpha activity in the parietal and central regions suggests better cognitive resource integration during rest (Benedek et al., 2014); increased frontal alpha activity may relate to more effective executive control and task inhibition mechanisms (Wöstmann et al., 2019). Meanwhile, significant activation of the beta1 band further supports superior information processing, task prediction, and motivational control (Devos et al., 2022). Regarding task performance, accuracy and reaction time in both congruent and incongruent conditions were significantly correlated with alpha and beta power in multiple brain regions, especially in the frontal, parietal, occipital, and temporal areas, showing a complex and stable functional mapping relationship between frequency bands and brain regions. Furthermore, under the congruent condition, theta waves in FP1 and FP2 were negatively correlated with accuracy, while alpha2 and beta1 waves in multiple regions were positively correlated with accuracy, indicating that effective cortical inhibition and executive activation are equally important for tasks with low conflict. The relationship between EEG indices and behavioral performance under the incongruent condition was generally consistent with the congruent condition, further validating the critical role of alpha1, alpha2, and beta1 power values (Misselhorn et al., 2019; Pscherer et al., 2023). Under conditions involving complex inhibitory tasks, the degree of activation in these frequency bands more significantly predicted task accuracy, indicating that these frequency bands are not only reflections of cognitive ability but also active indicators of information integration in cognitive control processes. In high cognitive load situations, the neural system requires more efficient synchronization mechanisms to maintain correct task execution (Xiang et al., 2022). This study identifies common EEG indices that are significantly correlated with both cognitive and inhibitory functions in the elderly with cognitive impairment. Key indices include alpha1 in C3, C4, P3, P4, O1, O2, F7, F8, T5, T6; alpha2 in C3, P3, P4, F8, T5; and beta1 in P3, F7, F8. These brain regions are widely involved in cognitive control networks and attention regulation pathways, and enhanced activity in different frequency bands represents superior information integration, conflict monitoring, and behavioral inhibition. From a neurophysiological perspective, the activation of alpha and beta frequency bands is believed to reflect functional coordination among higher cortical areas, with the level of synchronization determining the neural system’s flexibility and efficiency when facing interference and task switching (Sauseng et al., 2005). In particular, the enhancement of alpha activity in the fronto-parietal cortex is considered a core feature of improved cognitive control, while the enhancement of beta1 waves may reflect increased consistency in cortical-cortical transmission pathways within the executive network (Sacchet et al., 2015). These results not only reveal the neurophysiological mechanisms underlying cognitive impairment in older adults but also provide theoretical support and potential biomarkers for the future development of EEG-based early identification and intervention strategies.

In addition, this study further reveals the close behavioral and neuroelectrophysiological associations between muscle strength and inhibitory function, providing new evidence for understanding the interaction mechanisms between muscular health and cognitive control abilities. Behaviorally, muscle strength was significantly positively correlated with accuracy and significantly negatively correlated with reaction time under both congruent and incongruent task conditions. This indicates that older adults with greater muscle strength not only responded more quickly but also more accurately during inhibitory tasks, demonstrating more efficient cognitive inhibition. These findings align with the recently proposed “muscle-brain axis” theory, which posits that skeletal muscle, as a peripheral organ, can influence central nervous system function through multiple pathways such as neurotransmitter regulation and the secretion of metabolic factors (Arosio et al., 2023). Greater muscle strength may not only reflect a healthier physiological state but also indirectly support the structural and functional integrity of the prefrontal cortex, thereby enhancing executive function performance (Cai et al., 2023). EEG results further revealed a positive correlation between muscle strength and neural activity in specific frequency bands. Specifically, power in the alpha1 and alpha2 bands increased with muscle strength in regions such as the right frontal (F8), parietal (P4), and central (C4) areas, suggesting that muscle strength may modulate neural network activity in these areas to support more efficient inhibitory control. The enhancement of alpha activity in the right frontotemporal region may reflect better response inhibition control, while increased parietal alpha activity supports processes of information integration and conflict resolution (Bönstrup et al., 2015). The enhanced activity in these frequency bands among individuals with greater muscle strength suggests more efficient and stable neural functioning in the regulation of inhibitory responses (Manor et al., 2023). Furthermore, group-level analyses revealed gender differences in the effect of muscle strength on inhibitory function. Participants with normal muscle strength outperformed those with low muscle strength in terms of accuracy and reaction time under congruent conditions and showed higher activation levels of alpha1 and alpha2 in relevant brain regions. Notably, activation was most pronounced in the right frontal and parietal areas (e.g., F8, P4) among males with normal muscle strength, suggesting that this group may possess superior neural plasticity or advantages in metabolic regulation. These gender differences highlight the potential protective role of muscle strength in maintaining neural system function, which may be influenced by factors such as hormone levels, lifestyle, and neural regulation mechanisms . At the neurochemical level, muscle strength may influence cognitive function by promoting synaptic plasticity. Previous research has shown that skeletal muscles release various myokines—such as brain-derived neurotrophic factor (BDNF), insulin-like growth factor 1 (IGF-1), and interleukin-6 (IL-6)—during muscle activity or resistance training, which are closely associated with neural plasticity (Kostka et al., 2024). These factors can cross the blood-brain barrier and promote synaptogenesis and long-term potentiation (LTP) in the hippocampus and prefrontal cortex, enhancing neuronal connectivity and thereby improving cognitive performance. Additionally, dopamine, a key neurotransmitter involved in executive function and motivational behavior, may also be modulated by related metabolic pathways (Anzalone et al., 2012; Liu and Kaeser, 2019). Therefore, muscle strength may exert its beneficial effects on cognitive function through the regulation of neurotrophic factors such as BDNF and the dopaminergic system. In summary, this study emphasizes that muscle strength is not only an external indicator of physical health but also a crucial physiological basis for regulating cognitive control abilities. The activation of specific brain regions in the alpha frequency band may constitute a neural pathway through which muscle strength modulates inhibitory function. Integrating mechanisms of synaptic plasticity and neurotransmitter regulation, these findings offer a novel muscle-neural interaction perspective for understanding cognitive decline in older adults and provide theoretical support and potential biomarkers for developing physical fitness-based (e.g., resistance training) intervention strategies.

This study has several limitations:1.The study adopted an observational design, in which relevant indicators were measured at a specific time point, thereby only clarifying the cross-sectional relationships among muscle strength, inhibitory function, and cognitive function. Future studies could employ randomized controlled trials to explore causal relationships and specific mechanisms between these variables;2.This study did not include physical activity–related variables in the analysis. Future research could incorporate kinematic indicators to explore multivariable relationships and provide evidence for improving muscle strength and delaying cognitive decline in older adults;3.Participants in this study were grouped solely based on their MoCA scores, without stratifying by other demographic variables. Future studies could expand the sample size and examine differences among subgroups, as well as the relationships among variables across different demographic categories.4. In this study, *p*-value correction was conducted for only six EEG frequency bands, which may underestimate the spatial specificity associated with different electrode sites. Future research will aim to expand the sample size and incorporate multimodal data. By applying stratified correction methods and brain network modeling, we intend to investigate the mechanistic role of EEG biomarkers—across different electrode locations and frequency bands—in the interrelationship among cognitive function, inhibitory control, and muscle strength in older adults with cognitive impairment.

## Data Availability

The raw data supporting the conclusions of this article will be made available by the authors, without undue reservation.
